# Conundrum of IP_6_

**DOI:** 10.1098/rsob.150048

**Published:** 2015-11-18

**Authors:** Ivana Vucenik

**Affiliations:** Department of Medical and Research Technology, University of Maryland School of Medicine, 100 Penn Street, Baltimore, MD, USA

## Abstract

Here are comments on the recent paper on the determination of inositol hexaphosphate (IP_6_) in human plasma and on its efficacy.

Dear Editor(s),

Wilson *et al.* [1] describe a novel method for determination of inositol phosphates in biological fluids and report that, in contrast with previous reports from various other investigators including Grases and co-workers [2–4], they could not detect inositol hexaphosphate (Ins*P*_6_ or IP_6_ in short). This is in agreement with the previous report by Dr Irvine and co-workers [5]. While I cannot comment on the methodology owing to its novelty, I, however, noted that the authors have not provided any information about the humans whose plasma and urine were tested. Grases and co-workers [2–4] have conclusively and reproducibly demonstrated that in both experimental animals and human volunteers, the level of Ins*P*_6_ is very low to undetectable if the animals or humans are on an Ins*P*_6_-deficient diet. However, following a dose of Ins*P*_6_ supplement or diet containing high Ins*P*_6_, as in typical Mediterranean diets, substantial amounts of Ins*P*_6_ are detected in the plasma, urine and other fluids [2]. Therefore, it would be useful to know the dietary habits of the subjects whose plasma and urine were tested; this is, a part of a good and well-planned research design. Were they eating an Ins*P*_6_-poor diet or Ins*P*_6_-sufficient diet? A typical ‘fish and chips' or ‘meat and potato' diet is not likely to have any Ins*P*_6_.

As if that is not a serious enough flaw in the study design and hence the paper, the authors go on to conclude that since *they* could not detect Ins*P*_6_ in their samples of plasma and urine, therefore, Ins*P*_6_ should not be used as a dietary supplement … an issue that is totally irrelevant to the subject matter of the report and not supported by the data in the paper. In support of their conclusion, the authors draw a straw-man argument about the mineral bioavailability of Ins*P*_6_ based on outdated information. However, they have not provided any data of their own to support that Ins*P*_6_ is not safe or biologically ineffective in various diseases reproducibly demonstrated in the literature. Nor have they cited any published study unequivocally demonstrating the toxicity of pure Ca–Mg–Ins*P*_6_ as it occurs naturally and as dietary supplement. I am not aware of any study that refutes the various biological actions of Ins*P*_6_. On the contrary, impressive biological effects and multiple mechanisms of action for Ins*P*_6_ have been reported by different research groups all over the world. Its anti-cancer effect was found to be associated with the modulation of multiple genes involved in immunity, Wnt and IGF pathways, Akt, PI3 kinase, PKC signalling pathways and telomerase activity in leukaemia, breast and prostate cancer [6–9]. Anti-proliferative effects, induction of apoptosis and differentiation, and angiogenic effects were reported [6–10]. In addition to anti-cancer effect, other beneficial effects for human health, such as management of the Alzheimer's disease [11], and obesity and diabetes [12] have been described, highlighting even more mechanisms of action. Clinical studies show that patients on Ins*P*_6_+inositol supplement enjoy better quality of life in addition to remarkable regression of tumours [13–15]. Therefore, I would urge the authors to specifically address these two issues in their response (i) provide their data or published study unequivocally demonstrating the toxicity of pure Ca–Mg–Ins*P*_6_ and (ii) show the data or reference that it is not biologically active.

To the best of my knowledge, lifetime experiments with pure Ins*P*_6_ in rodents and well-designed human studies have not demonstrated any mineral deficiency or toxicity. A common sense question: Is the menace of cancer, kidney stone and other diseases any less than the hypothetical (and unsubstantiated) putative deficiency of cations that can be easily corrected?

There are other flaws in the paper that though may appear minor do, nevertheless, reflect poorly on the report and the authors' credibility in culling scientific data, e.g. Eiseman *et al.* [16] studied the pharmacokinetics in *mice* and not rats as described; the metabolism in the two species are different.

Finally, making conclusions and recommendations that are not supported by data and are at variance with logic, may erode public trust in science. Because the field of inositol phosphates and the use of IP_6_ in human diet have strongly polarized and sharply divided scientists, an open, healthy discussion, and some critical evaluations are needed.
